# Sharing individual participant data: through a systematic reviewer lens

**DOI:** 10.1186/s13063-021-05787-4

**Published:** 2022-02-21

**Authors:** Larysa H. M. Rydzewska, Lesley A. Stewart, Jayne F. Tierney

**Affiliations:** 1 MRC Clinical Trials Unit at UCL, Institute for Clinical Trials and Methodology, 90 High Holborn, London, WC1V 6LJ UK; 2 Centre for Reviews and Dissemination, University of York, York, YO10 5DD UK

**Keywords:** Data sharing, Systematic review, Clinical trials

## Abstract

An increasing prevalence of data-sharing models, aimed at making individual participant data (IPD) from clinical trials widely available, should facilitate the conduct of systematic reviews and meta-analyses based on IPD. We have assessed these different data-sharing approaches, from the perspective of experienced IPD reviewers, to examine their utility for conducting systematic reviews based on IPD, and to highlight any challenges. We present an overview of the range of different models, including the traditional, single question approach, topic-based repositories, and the newer generic data platforms, and show that there are benefits and drawbacks to each. In particular, not all of the new models allow researchers to fully realise the well-documented advantages of using IPD for meta-analysis, and we offer potential solutions that can help improve both data quantity and utility. However, to achieve the “nirvana” of an ideal clinical data sharing environment, both for IPD meta-analysis and other secondary research purposes, we propose that data providers, data requestors, funders, and platforms need to adopt a more joined-up and standardised approach.

## The current clinical data sharing landscape

The sharing of clinical trial data can increase transparency, improve understanding of individual trials, and facilitate the re-use of the data for secondary research, including meta-analyses of individual participant data (IPD meta-analyses) in particular [1]. These have been described as the gold standard of systematic review [2] because they can improve the completeness and quality of data and the breadth and flexibility of analysis [3–6]. Notwithstanding the recent intensification of activity on this subject, whilst the concept of sharing IPD may be evolving, it is certainly not novel. Indeed, meta-analyses involving the central collection, checking and analysis of IPD, have been around since the 1980s [7, 8]. We present an overview of the range of different data-sharing models, as well as the traditional collaborative approach, and show that there are benefits and drawbacks to each. In particular, not all of the new models allow researchers to fully realise the well-documented advantages of using IPD for meta-analysis, and we offer potential solutions that can help improve data quantity and utility.

### The traditional collaborative approach to accessing IPD from trials

#### Single-question IPD meta-analysis

Traditionally, IPD meta-analyses have usually been carried out by a core research team, who build collaborations with trial investigators to gain access to IPD from all the trials relevant to a specific research question [9] (Table 1). Usually, a clearly defined set of variables, required to address this research question, are collected centrally. These will include information relating to the trial participants’ disease or condition, the treatments and tests they have received, and the outcomes collected. These data are standardised and checked, then verified with the trialists prior to analysis, and the results are usually published on behalf of the whole collaborative group [3, 5, 10].

However, there are some drawbacks to this traditional approach. For example, although it can provide full, direct access to the relevant IPD, the process of building such partnerships, preparing data use agreements, and sourcing, collecting, checking, and analysing the IPD can be much more lengthy, complex, and resource-intensive than it is for systematic reviews based on aggregate or summary data [4, 5]. It may also be difficult to locate the appropriate responsible party(ies) who can grant agreement to share the data and/or find out where the required data are held. Furthermore, although data use agreements are now much more common, in our experience, these are becoming increasingly more difficult to negotiate between data providers and recipients [9]. Ironically, although trial investigators are now much more familiar with, and amenable to, requests for sharing their data, the development of the data use agreements invariably involves not just trialists and researchers, but also legal teams from both sides, which can lead to a prolonged and complex contractual undertaking. Moreover, the ability to re-use or share the IPD with other researchers, in order to address new questions that may arise, is often inhibited by the need to obtain additional permissions from the original trial investigators.

#### Topic-based IPD meta-analysis repositories

Topic-based repositories aim to bring together IPD from all trials within a particular health-care area, with the specific intention of addressing multiple questions, and in some cases, the subsequent re-use by the research community to address new clinical questions [9] (Table 1). These may be developed either retrospectively or prospectively.

Retrospective topic-based repositories represent the retrospective amalgamation of IPD from multiple single-question meta-analyses that may be expanded over time leading to the establishment of a topic-based database. For example, the rich collection of data gathered by the Early Breast Cancer Trialists’ Collaborative Group (EBCTCG) [11] since the 1980s as part of a seminal series of IPD meta-analyses (assessing, e.g. tamoxifen [7], polychemotherapy [12], and radiotherapy [13]) and the ACCENT database of colorectal cancer trials [14]. Such databases can subsequently be utilised to address a further variety of key clinical questions, such as treatment efficacy questions [15–17], the investigation of potential prognostic factors [18], and identification of surrogate endpoints for overall survival [19].

Examples of more forward-looking repositories include ICECaP, which has evaluated surrogate outcomes in localised prostate cancer, but aims to include newer trials as they become available to validate these outcomes [20]. Likewise, ARCAD [21] aims to pool data from completed colorectal cancer trials, to identify optimal trial endpoints [22], and to investigate predictive and prognostic factors [23] in this setting.

Prospectively designed topic-based repositories take this approach a step further by mapping out the trial landscape, before most have completed and reported, to address a series of questions (both known and as yet unknown) within a specific topic area. One such example is the STOPCAP M1 repository of trials conducted in metastatic hormone-sensitive prostate cancer [24]. Using a prospective approach allows researchers to anticipate upcoming, clinically relevant, and important questions as the treatment paradigm evolves, and direct engagement with investigators facilitates the conduct of prospective meta-analyses of aggregate data that can guide the strategic collection of IPD [25]. As with retrospective topic-based repositories, the data are also being used to identify and validate early surrogate outcomes and prognostic factors, and data are also available to the wider research community, under a moderated access model, for further clinical and methodological research.

The topic-based approach can provide the triple efficiency of ensuring that the clinical data collated are harmonised, thereby removing that burden from research teams wishing to use the data subsequently; allowing trial investigators to comply with data-sharing policies; and reducing the need for trials teams to meet multiple and varied requests for data. Moreover, repeated use of the data by different research teams to answer key clinical questions, maximises the value of these data, whilst minimising research waste. However, as this broadly uses the same approach to gain access to and manage the IPD as the single-question model, it invariably similarly suffers from the same drawbacks, and the process can still be both time and resource intensive.

### Accessing IPD via generic data hosting and sharing platforms

The numerous recent calls for increased clinical data sharing from a wide variety of sources [26–32] have also led to the development of a raft of new initiatives, based on a more generic data-sharing model. As these have developed contemporaneously, but somewhat independently [33], they offer varying levels of access to the underlying data (Table 1).

At one end of the spectrum, fully anonymised IPD may be available to download directly, without restriction (e.g. the freeBIRD database [34]). At the opposite end, the repository software has been designed to permit users to “take the analysis to the data”; in other words, the data are interrogated remotely, without the researcher ever having physical access to it (e.g. DataSHIELD [35]). More commonly, access is via data-hosting repositories such as the National Cancer Institute’s NCTN/NCORP Data Archive [36] or data-sharing platforms such as Clinical Study Data Request (CSDR) [37] or the Yale Open Data Access Project (YODA) [38]. The latter do not normally host data, but rather provide a mechanism to facilitate access to the IPD. Access is usually subject to the approval, often by an independent scientific committee, of a research proposal, and the research team signing up to a standard platform-specific data use agreement. Thereafter, researchers can either download the data, or the IPD may only be made available within a secure area of the platform, sometimes referred to as a “locked-box” approach. A more recent addition to the data-sharing arena is Vivli, which was launched in 2018 as both a global platform and a general clinical data repository [39], and therefore acts as either the host of, or to facilitate access to, clinical trial data across multiple platforms.

These novel initiatives heralded the potential to make gathering IPD quicker and easier, as they provide a useful mechanism for more easily locating IPD from relevant individual studies, which has often been a key problem with the traditional approach. Also, simple “off the peg” data use agreements potentially allow much more timely access to the requested data, as long as the agreement template enables the research team to achieve the specific aims of the proposed project (otherwise bespoke data use agreements may be necessary); although timings may vary between platforms and even between data providers within platforms. However, it is still unclear whether these new approaches are ultimately any quicker or better compared with the traditional collaborative approach [1, 43]. For example, the majority of these novel generic data sharing platforms simply provide access to a limited and ad-hoc set of individual trials, such as those conducted by a particular company or institution, to comply with data-sharing requirements. There can also be considerable variability in both the quantity and quality of the data that are deposited in these platforms, and in some cases, the datasets may have been heavily redacted. All of these issues can render the data unsuitable for a systematic, reliable, and thorough investigation of a specific research question.

Another consideration is that many IPD meta-analysis projects will still require data from older and/or non-industry or academic trials, which are currently under-represented within the newer data-sharing initiatives. For example, from a CSDR metric report, of the 3374 studies on the platform at that time, only 10 of these were from academic funders [43] and in a recent survey of researchers, funded primarily by one of four main funders (Wellcome Trust; Medical Research Council; Bill and Melinda Gates Foundation; and Cancer Research UK), of those who had shared their data, only 17% had stated that the data would be made available via a repository in their sharing statements [44]. Therefore, it is likely, that for these trials, IPD will continue to be sought directly from trial investigators for some time to come.

**Table 1:** Examples of types of data-sharing models

Data-sharing model	Level of access	Mode of access	Type of data	Level of collaboration with original trialists	Examples
**IPD released directly to data requestor**					
**Single-question IPD meta-analysis**					
Usually to address one or more specific research questions	Negotiate provision of IPD from trialists	IPD provided directly to data requestor	Usually pseudonymised	Usually fully collaborative	Single-collaboration IPD reviews [40, 41]
**Topic-based repository**					
*Retrospective:* Assembly of multiple existing IPD databases within a topic area to address new questions. May include new trials as they become available	Negotiate provision of IPD from trialists	IPD provided directly to data requestor	Usually pseudonymised	Collaborative	ACCENT [14]; ARCAD [21]; EBCTCG [11]; ICECaP M0 [20]
*Prospective:* Plan to assemble IPD from all trials within a topic area, whilst most of the trials are still ongoing or yet to report, to address planned questions or new questions arising					STOPCAP M1 [24]
**IPD released to requestor via data platform**					
*Data available on demand with unrestricted access*	Data available to download online (usually only registration is required)	IPD provided directly to data requestor; usually via direct download	Usually fully anonymised	Not usually collaborative	freeBIRD [34]; International Stroke Trial (IST) database [42]
*Moderated access*	Subject to the approval of a formal research proposal	IPD provided directly to data requestor	May be fully anonymised or pseudonymised	Potential to be collaborative	CSDR [37]; YODA [38]; VIVLI [39]; NCTN/NCORP [36]
**IPD accessed/analysed within host platform**					
*Moderated and restricted (“locked-box”) access*	Subject to the approval of a formal research proposal	IPD available only within secure environment	May be fully anonymised or pseudonymised	Not usually collaborative	CSDR [37]; YODA [38]; VIVLI [39]; NCTN/NCORP [36]
**Remote data interrogation**					
*Analyses taken to the data*	Subject to approval of a formal research proposal	IPD interrogated remotely; question(s) and/or analysis software provided by the data requestor	Not applicable— no direct access to data	Not usually collaborative	DataSHIELD [35]

## The utility of different the data-sharing approaches for IPD meta-analysis

### Improving the completeness and quality of data

In the traditional collaborative and topic-based IPD approaches, relevant data from all eligible trials, all randomised participants, and all relevant outcomes are usually sought, along with extra follow-up (if relevant) (Table 2). This means data from unpublished trials, participants and outcomes can be included, which can alleviate potential reporting and data availability bias, and other limitations associated with meta-analyses based on published aggregate data [5, 45] and allow long-term treatment effects to be examined [3–5].

**Table 2:**
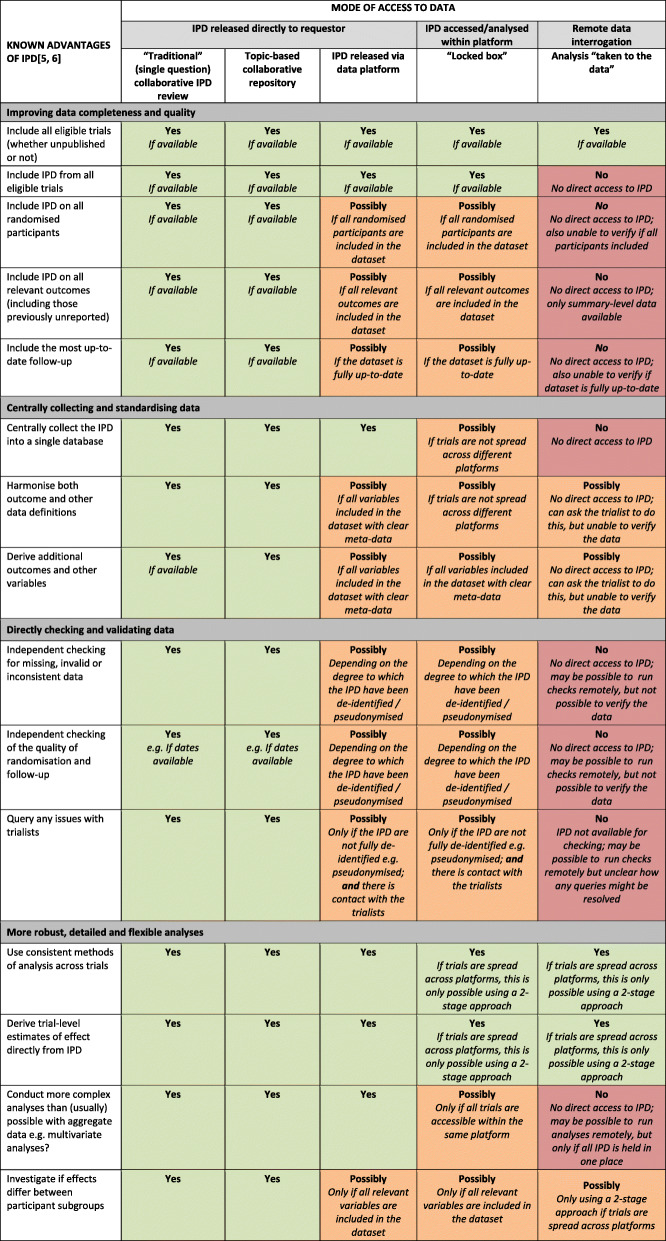
Advantages of the IPD approach under different data-sharing models

Instead, data deposited in platforms may only include a very specific subset of the clinical data collected within the original trial, such as those participants included in the published analyses, or those that received per-protocol treatment, or data from just those outcomes that have been previously reported in a journal article, rather than all the participants or all relevant variables required for a planned meta-analysis. Also, depending on when a particular dataset was submitted to the platform, it may not be based on the most up-to-date follow-up. IPD that are limited in such ways may introduce some of the biases usually associated with published aggregate data.

### Centrally collecting and standardising data

For IPD meta-analysis, gathering the data together into a single space allows for variable definitions to be standardised as far as possible across all trials, for uniform coding of the data, and the derivation of any additional outcomes or other variables of relevance to the analysis [3–5, 10], (Table 2). In the traditional approach, much of this is often done by the data providers, or by the central research team with input from the original investigators (who understand the data best). This standardisation then makes it possible to readily combine data across trials in a meta-analysis, and to interpret the results more easily.

In contrast, in many of the newer data-sharing platforms, where data are accessed remotely within separate, secure “locked box” environments, the relevant trials may also be dispersed across multiple platforms. This can impede the effective harmonisation of the datasets and subsequently make it difficult or impossible to derive any further variables, outcomes, or other data definitions. This is less of an issue if the data are released directly to the reviewer through the platform, provided the appropriate meta-data are available. However, without input from the original trialists this could still prove difficult. In addition, even if it is possible to download the data directly from one platform, the “off the peg” data use agreement put in place to access the data will likely prohibit the subsequent uploading of that data to another repository, where data are only accessible via the “locked box” model.

### Directly checking and validating data

Direct access to IPD allows the research team to check the completeness, validity, and consistency of the data and to query any anomalies with trialists [3–5, 10] (Table 2), which also helps them to better understand the data [3]. In addition, the original trialists can help to clarify aspects of trial design and conduct to inform risk of bias assessments of the included trials [46], which can be enhanced by direct checking of the IPD, for example, to check the randomisation process [3, 5, 10].

However, within the newer, secure, environments, the degree to which the data have been de-identified or redacted will determine the extent to which the reviewer can directly check and validate the IPD. For example, if no dates are provided, it will not be possible to determine the internal consistency of, for example, time-to-event outcomes, or to thoroughly check the integrity of randomisation [3, 5]. Furthermore, even if such checks are possible, if a direct link to the trialists cannot be established via the platform, it will not be possible to address any queries and/or to verify the data. In the most extreme data-sharing scenario, where IPD are only available for remote data interrogation, although it might be possible to ask the trial teams to run such checks, they may be unwilling to do so, and this cannot compare with the scrutiny of the underlying data by an independent group. Indeed, for data that are only accessible remotely, requesting access to the trial clinical study reports [47, 48], instead of the IPD, may actually provide more transparency.

### More robust, detailed, and flexible analysis

The central collection and harmonisation of all the IPD into a single database not only permits the use of consistent methods of analysis across all trials, and for estimates of effect to be derived directly from the data, but also facilitates a more in-depth analytical approach [5, 6]. This includes the investigation of any potential variations in treatment effect across different types of participant and the use of more complex models [5, 6] (Table 2). This is only fully possible when data from all trials are released directly to the reviewer and collected in a single space. If one or more trials can only be accessed either within separate platforms, or remotely, even if the relevant software is available or can be brought into the platform, the IPD will need to be distilled into aggregate data for analyses. Hence, the choice of potential analyses will be restricted, and for example, only a two-stage, rather than a one-stage, meta-analysis model for both overall effects and interactions would be feasible. This would also render development of IPD prediction models near impossible and other complex or multivariate analyses at the participant level would not be possible at all.

## Discussion

### Challenges of the different data-sharing models

In systematically evaluating the current clinical data-sharing models against the known advantages of conducting reviews and meta-analyses based on IPD [5, 6], as listed in Table 2, we have found that a number of challenges remain. In fact, none of the individual models described in this paper fully allows researchers to quickly and efficiently locate, centrally gather, standardise, check, and flexibly analyse all the relevant data to address a specific question.

Whilst there has been a steady stream of publications addressing the importance of sharing clinical data to promote transparency, validate study findings, and conduct secondary research [49], much of the the focus has been on how to make data available [50], meta-data standards [51], and ethical considerations [52], rather than consideration of maximising the value and utility of that data for IPD meta-analysis. This is reinforced by a survey of CSDR users suggesting that “analysing data behind a firewall is burdensome and inadequate for meta-analyses of patient-level data” [37], and more recently by members of CSDR’s Independent Review Panel (IRP) who have acknowledged challenges to the sustainability of the CSDR model given that data access has been considerably lower than originally anticipated [43]. Broader evaluation of the current data-sharing landscape, for example, to assess either the suitability of different platforms for hosting IPD [33], or their ease of use for researchers wishing to access data [53] have also drawn attention to heterogeneity across existing platforms, and have highlighted the fact that increased availability of data does not necessarily lead to faster access to that data [1, 43]. Furthermore, with data largely made available via generic platforms to comply with policy, this potentially creates silos of data that may never be requested and utilised by others [37, 43]. Thus, the value of this blanket approach seems unclear, particularly given that preparing data for sharing has considerable resource implications.

### How can data providers and data requestors help to maximise the use and value of the clinical data?

The current data-sharing landscape is presently in a state of flux, with all platforms and repositories continually evolving, and therefore, there is the potential to eventually create an ideal data-sharing scenario. We would argue that from the systematic reviewer perspective, improving the current data-sharing landscape not only requires increased visibility and accessibility of all data, but also the ability to gather together all trials relevant to a specific question into a single space, with the IPD harmonised as far as possible across the included trials. In order to achieve this, a more forward-thinking and cooperative approach will be required:

Ideally, for all new trials being designed, trialists should aim to prospectively obtain consent from participants for the re-use of their data after the trial has been completed. In addition, when preparing participant data for sharing; as far as possible these should be suitably detailed and standardised according to the norms for the clinical area. If a minimum level of standardisation is not already in place, especially for composite and/or complex baseline characteristics and outcomes, then there may still be a limit to the level of harmonisation possible at this stage. Here, the topic-based approach could play a key role in informing standards for the deposition and curation of data within a particular health-care area, for example, by highlighting the key variables to include, and outcome variables could be informed by core outcome sets (if they exist) for trials within a specific disease area or condition [54]. Also, in those cases where data are being sought directly from the original investigators, it is important that data use agreements are simplified as far as possible, whilst continuing to appropriately govern the flow and use of data and protect the privacy of participants; as per the agreements for some of the newer platforms.

Systematic reviewers (or other data requestors), who find that data have already been deposited in a platform or repository, but are insufficiently detailed or complete, should interact directly with trial teams to seek the additional relevant data. Although the main data-sharing platforms, e.g. Vivli, CSDR, and YODA, do have channels through which such enquiries can be made, in some cases, this may only be to a third party that has prepared the data for deposition, who may not understand the data as well as the original trialists. Therefore, additional efforts may still be required to contact the relevant trial personnel directly. Ideally, this should be aligned to their IPD meta-analysis or topic-specific data dictionary, which would not only provide the data needed to conduct a specific IPD meta-analysis, but would also increase the utility of the data to other research teams who might access it subsequently. Similarly, if the data have not yet been deposited, then researchers should liaise with the trialists in advance of preparation of the data. In both cases, this could potentially reduce the need for data providers creating multiple versions of a particular clinical dataset, which can be an issue for key clinical trials. Furthermore, where additional data are requested for trials accessed via a generic data platform, an entirely new dataset is usually uploaded to the platform by the original trial team. However, it is less clear how any changes, identified through the standard IPD checking process, and subsequently made to the (e.g. downloaded) datasets accessed by researchers, are managed. Mechanisms need to be in place across all repositories to ensure that any subsequent data requestors can access these same ‘corrected’ trial datasets.

However, even if the quality and quantity of the data made available for sharing are improved, facilitating the conduct of IPD meta-analysis will still require better interoperability and flexibility across the different data-sharing platforms, for example by allowing data to be downloaded directly, or to allow data from different repositories, and data obtained directly from trial investigators, to be brought together. The Vivli platform [39] has set an example and started to address this issue by acting as a “bridging” platform to allow researchers to collate data from different repositories, but this is currently limited to those data providers/platforms that are signed up to Vivli, leaving much scope for improvement.

It is also important to remember that the concept of sharing IPD is not novel and has been helping to improve patient outcomes worldwide for decades [55]. Whilst it is understandable and important that safeguards need to be in place to govern the appropriate and secure sharing of clinical data, there is also little point in sharing data that is not fit for the research purpose for which it is being requested. Therefore, with suitable data use agreements in place, to minimise risk and ensure that data are only accessed by specific researchers with appropriate skills, it should be possible to share de-identified, rather than fully anonymised or heavily redacted data. Given that many funders are mandating deposition, they could also have a role to play in ensuring that deposited data are fit for re-use. Another consideration for funders is whether sharing of all clinical trials should be mandated, or whether an “on-demand” approach would be more appropriate, whereby data are prepared for sharing when the first approved access request is made, to ensure that all important data are shared, whilst limiting the burden on trial teams.

A recent scoping study, which aimed to explore different data sharing approaches, and their impact on research outputs, demonstrated that there is still a lack of empirical data to assess the link between intended and actual data-sharing [56]. Therefore, we strongly believe that any future efforts to address this evidence gap should consider the issues raised within this paper.

## Conclusions

To achieve the “nirvana” of an ideal clinical data sharing environment for IPD meta-analysis and other secondary research purposes, data providers, data requestors, funders, and platforms must adopt a more joined-up and standardised approach.
